# Hypothesized Mechanisms Through Which Exercise May Attenuate Memory Interference

**DOI:** 10.3390/medicina56030129

**Published:** 2020-03-14

**Authors:** Lindsay K. Crawford, Hong Li, Liye Zou, Gao-Xia Wei, Paul D. Loprinzi

**Affiliations:** 1 Exercise & Memory Laboratory, Department of Health, Exercise Science, and Recreation Management, University of Mississippi, Oxford, MS 38677, USA; 2 School of Psychology, Sichuan Normal University, Chengdu 610101, China; 3 Exercise & Mental Health Laboratory, School of Psychology, Shenzhen University, Shenzhen 518060, China; 4 Key Laboratory of Mental Health, Institute of Psychology, Chinese Academy of Sciences, Beijing 100101, China; weigx@psych.ac.cn

**Keywords:** cognition, exercise, proactive memory interference, retroactive memory interference

## Abstract

In this paper we introduce a mechanistic model through which exercise may enhance episodic memory, specifically via attenuating proactive and retroactive memory interference. We discuss the various types of memory, different stages of memory function, review the mechanisms behind forgetting, and the mechanistic role of exercise in facilitating pattern separation (to attenuate memory interference).

## 1. Introduction

**Figure 1:**
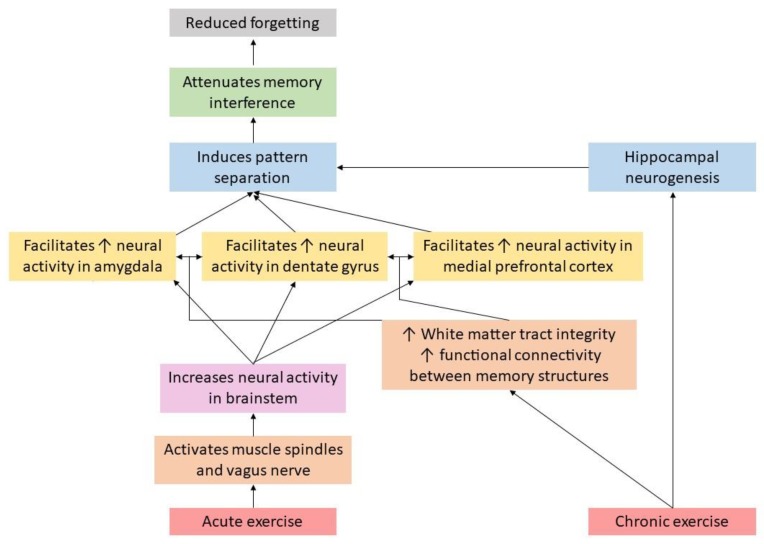
Mechanisms through which exercise may attenuate memory interference.

Given the importance of enhancing and maintaining memory function, the focus of this review is to discuss the potential role that physical exercise may play in minimizing cognitive memory interference, which in turn, may help to facilitate memory function. Further, we discuss a *hypothetical* model detailing potential mechanisms through which physical exercise may attenuate cognitive memory interference, which is displayed in Figure 1. This model, which is unpacked throughout this paper, suggests that acute physical exercise may increase neural activity (via muscle spindle [1,2,3] and vagus nerve activation) in several key memory related brain structures (e.g., amygdala, hippocampus, and medial prefrontal cortex), which, in turn, may help facilitate pattern separation, and ultimately, attenuate memory interference. Pattern separation refers to the ability to distinguish target information from other similar information (which often creates competition upon retrieval). For example, if an individual was instructed to learn a list of words which was comprised of types of fruits and vegetables, but they were not told this, they may be able to distinguish between the material independently by cognitively “separating” it in their minds into the subcategories of fruits and vegetables. On the other hand, and as displayed in Figure 1, chronic physical exercise may increase the functional connectivity across these key brain structures (via increased integrity of white matter tracts connecting these structures), as well as increase hippocampal neurogenesis, collectively, which may help attenuate memory interference via hippocampal pattern separation. Where appropriate, we discuss these pathways among both animal and human studies. We then conclude this paper with recommendations for future research on this emerging line of inquiry. 

## 2. Types of Memory

**Figure 2:**
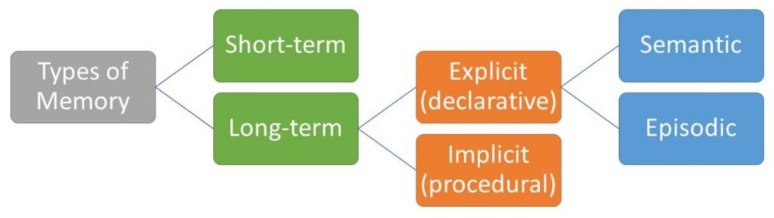
Types of memory [8].

There are a multitude of memory types, including non-declarative (e.g., implicit memory, procedural memory) and declarative memory (see Figure 2) [1]. Implicit memory may occur subconsciously, not requiring conscious thought after a learning period (e.g., driving, walking, etc.). Declarative memories are consciously formed, where semantic memory refers to the retrospective recall of non-contextual information. In contrast, episodic memory involves the retrospective recall of information that is contextually bound into a temporal-spatial context. That is, episodic memory involves the integration of what-where-when aspects of a memory and is considered autobiographical [4]. Research on memory interference, when specifically utilizing word lists, may be considered episodic memory as it can address the what, where, and when aspect of episodic memories. See Tulving’s conception of long-term memory for further readings on episodic memory [4,5,6,7].

## 3. Episodic Memory Stages

Forming an episodic memory involves several stages, including encoding, consolidation, reconsolidation, storage, and retrieval [9,10,11]. Encoding occurs when a stimulus (i.e., new information) creates a memory trace, or engram, within the brain [12]. These memory traces are collections of neurons that are formed within key memory-related structures, such as the hippocampus [12]. During consolidation, the memory trace is stabilized, assimilating into previous long-term knowledge, making it more likely to survive [12]. The final stage, retrieval, occurs when stored memories are recalled. 

## 4. The Role of Exercise

Physical exercise, including both acute and chronic, has been demonstrated repeatedly to improve memory function [13,14]. In the case of memory improvement, we are specifically referring to exercise (planned and structured) compared to unstructured, physical activity. The narrative that follows will briefly highlight the mechanisms through which acute and chronic exercise influence memory function, particularly episodic memory function. Specifically, aerobic exercise has demonstrated to be an effective method of inducing neuroplasticity, although the mechanisms are not fully understood [15]. Evidence can be detected at the molecular, cellular, and systems levels. At the molecular level, aerobic exercise has been shown to alter the concentration of peripheral brain-derived neurotrophic factor (BDNF), insulin-like growth factor 1 (IGF-1), and vascular endothelial growth factor (VEGF), which are responsible for promoting neuroplasticity, brain development, and providing a vascular environment suitable for neurogenesis [15]. These alterations cause cellular changes in the brain, which are discussed further in the following sections [15]. 

## 5. Chronic Exercise

Exercise has been shown to produce structural and functional changes within the brain [16]. In human studies, structural changes are measured as increased hippocampal and prefrontal cortex (PFC) volume and white matter integrity, potentially mediated via neurogenesis, brain-derived neurotrophic factor (BDNF) [16,17] and other related mechanisms [18]. These neuronal changes lead to neuroplasticity, increased efficiency of BDNF uptake, and upregulated transcription and signaling cascades [15]. Increases in synaptic size and density are demonstrated in human and animal studies along with other structural changes that are not explained by novel or enriched environments [19,20]. As early as 1999, researchers demonstrated that wheel running in mice increased the number of new neurons (i.e., neurogenesis) within the hippocampus [19]. The connection between exercise and brain volume has also been identified in children using magnetic resonance imaging (MRIs), where VO_2_ max (maximal oxygen uptake) is positively associated with hippocampal volume [19]. Chronic aerobic exercise also induces increases in circulating BDNF (brain-derived neurotrophic factor), IGF-1 (insulin-like growth factor 1), and VEGF (vascular endothelial growth factor), all of which promote gliogenesis, neurogenesis, synaptogenesis, and angiogenesis [15]. These effects may mediate increases in grey and white matter volume, neural activity, and cerebral blood flow [15]. This may, in turn, increase synaptic plasticity, ultimately affecting episodic memory [15,21,22]. 

## 6. Acute Exercise

In experiments examining the role of acute exercise on memory of humans, walking, jogging, and stationary cycling are the most evaluated modes of exercise [13]. A recent meta-analysis demonstrated that walking may be the most effective mode to improve episodic short-term memory (e.g., immediate recall), while cycling may be most effective for improving long-term memory (i.e., recall longer than 2 minutes after exposure) [13]. Acute (i.e., single bouts) aerobic exercise has been found to positively affect long-term memory in the majority of experiments examined [13]. There is a positive trend towards acute (48% of studies) aerobic exercise improving short-term memory, however, these findings are not consistent across the literature [13]. For example, Hotting et al. found that an acute bout of exercise after learning did not improve the number of words recalled, but the high intensity exercise group forgot less vocabulary at a 24-hour follow up [23]. In a 2016 study, Etnier et al. found that different acute exercise intensities did not influence free recall, but did have an effect of long-term memory recognition [24]. Thomas et al., on the other hand, found that high intensity acute (45 minutes) exercise enhances long-term retention of information [25]. 

This inconsistency may be due to a variety of potential moderators, including age, sex, exercise mode, exercise duration, intensity of exercise, and fitness level of the participants [13,26,27]. Acute aerobic exercise has also been shown to increase levels of peripheral BDNF and VEGF, increase neurotransmitter concentration, and increase glucose and oxygen metabolism [15]. Acute exercise also increases cerebral blood flow, neural activity, and receptor activity, which may lead to increased neuroplasticity [15], and ultimately, enhanced memory function. Recent work demonstrates that acute exercise may induce neuroplasticity via I-BAR gene expression [28].

As we have reviewed elsewhere [29], acute exercise is likely to induce these neural responses by increasing neural excitability in various memory-related brain structures, such as the hippocampus and prefrontal cortex. Such effects likely occur from acute exercise-induced activation of muscle spindles [1,2,3] and afferent fibers of the vagus nerve, which will be detailed further in Section 11 below.

## 7. Memory Interference

Although studies that investigate the effects of exercise on memory are accumulating, considerably less research has focused on the effects of exercise on forgetting. Only a small amount of information we encode is consolidated and added to our long-term memory. Most information is lost—forgotten—either temporarily or permanently. There are a multitude of mechanisms through which forgetting occurs, including via passive and active mechanisms, which affect the integrity of memory engrams [30]. Passive forgetting occurs when memory traces, or engrams, decay naturally [30]. When parts of an engram are unable to respond to activation, it can become difficult to retrieve the memory [30]. The form of forgetting discussed herein, which is of central focus of this paper, is memory interference, which occurs when competing information occurs before, after, or during the encoding of target information [30]. Interference is more likely to occur if the competing information is similar, such as a list of semantically related words (e.g., doze, bed, slumber, dreams, and nap) [31,32]. 

**Figure 3:**
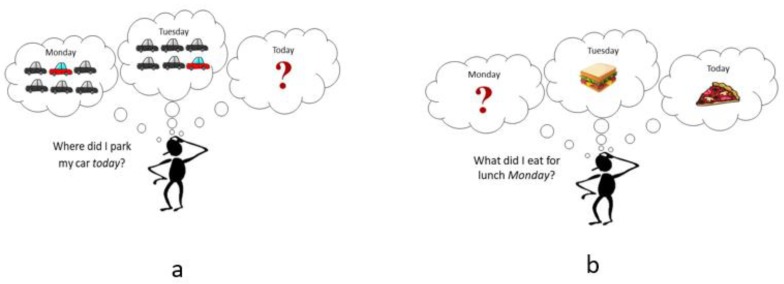
**(a)** An example of proactive memory interference. Not being able to recall where you parked your car today at work since you park in the same parking lot repeatedly. (**b)** An example of retroactive memory interference. Remembering what you ate for lunch today (Wednesday) and yesterday (Tuesday), but not being able to recall what you ate for lunch on Monday.

There are two main types of memory interference—proactive and retroactive. Proactive interference (PI) occurs when previously acquired knowledge interrupts the acquisition of new information. In essence, you have “old” information inhibiting the recall of “new” information (old → new). For example, calling a new student in your class by a previous student’s name. See Figure 3a for another example. This type of interference causes difficulty in learning and retaining new knowledge. 

Retroactive interference (RI) occurs in the opposite direction, new knowledge interrupts the recall of previously established knowledge (old ← new), which potentially disrupts memory consolidation [30]. Following the previous example, this would be calling a *previous* student by a *new* student’s name. See Figure 3b for another example. Retroactive interference is argued by some to occur more frequently and be more detrimental to memory when compared to proactive interference [33]. 

## 8. Consequences of Memory Interference

Research on memory interference began as early as 1892, when John A. Bergstrom conducted an experiment where participants sorted two decks of cards into two separate piles [34]. When the location for the second pile was subsequently changed, the sorting speed became significantly slower, demonstrating an interference effect (proactive) of the first set of rules on the new set of rules [34]. As such, interference is likely to occur when learned stimuli is associated with a new response [34,35]. 

There are multiple theories of memory interference. Some theorists argue that retroactive interference (new information impeding recall of old information) may be misidentified when a person has, in reality, simply forgotten the past material. Additionally, the temporal-distinctiveness theory focuses on how recollection of information depends on its isolation in psychological time [36]. This theory claims that discriminability and retrievability of to-be-remembered items is a direct function of their isolation in time. The closer the to-be-remembered items and competing items are in time—the more likely interference is to occur [36]. Another theory, the dual mechanisms of control theory, details that there are reactive and proactive control mode processes that respond to memory interference [37]. The reactive control mode is stimulated after detection of interference, whereas proactive control modes occur when information is maintained in a pre-emptive manner over a period of time, even before interference is introduced [37]. Both of these theories involve the temporal periods of target information and interfering information, as they play a significant role in memory function [36,37].

## 9. The Effect of Exercise on Cognitive Memory Interference

The literature on exercise and memory interference is scarce. In our first experiment on this topic, we conducted a between-subject study that addressed the effects of exercise on memory interference [38]. Participants were randomly assigned to one of four temporal periods: control, exercise before encoding, exercise during encoding, or exercise after encoding. There were two separate exercise protocols imposed, in which 88 participants completed a 15-minute bout of moderate intensity treadmill walking and another 88 participants completed a 15-minute bout of high-intensity treadmill exercise. The Rey Auditory Verbal Learning Task (RAVLT) was utilized as an assessment of proactive memory interference, which involved learning two separate lists of words. During a RAVLT protocol, participants learn a list (List 1) of 15 unrelated words over five different trials and are asked to repeat them. After the first five trials of list 1, another list (List 2) of 15 unrelated words are given and the participant must again repeat them. Performance on the second list was lower than performance on the first list, demonstrating evidence of proactive memory interference. The results demonstrated that high intensity exercise prior to memory encoding produced a non-significant tendency to attenuate a memory interference effect, as shown by a greater performance on the second list following exercise when compared to a non-exercise condition. 

Our second experiment also evaluated the temporal effects of exercise on attenuating a proactive memory interference effect [39]. This was a within-subject, counterbalanced design with four laboratory visits, including: a control visit, exercising prior to memory encoding, exercising during memory encoding, and exercising after memory encoding. The exercise comprised of 15-minutes of moderate intensity treadmill walking. The RAVLT was utilized as the memory interference task. The sample comprised of 24 young adults and results demonstrated that exercise occurring prior to the memory task had the strongest impact on improving memory performance of the second list of words, suggesting an attenuated proactive interference effect. 

Retroactive interference may impair long-term memory consolidation, therefore, we designed a study that focused solely on the ability of acute exercise to reduce this effect [40]. Three experimental studies were employed in this paper. Experiment 1 was a between-subject randomized control trial (RCT), which included a 15-minute bout of moderate intensity walking. Experiment 2 was a between-subject RCT, which included a 15-minute bout of high intensity jogging. Experiment 3 was a within-subject design that included a 15-minute bout of moderate intensity walking. For the memory task, the RAVLT was employed post exercise for the 112 participants. Based on our aggregate analyses, the pooled effect size (standardized mean difference was −0.35, 95% CI: −0.64 to −0.06) across the experiments was statistically significant (*p =* 0.01), providing evidence for acute exercise to attenuate a retroactive interference effect. These findings align with our earlier experiment [41].

We conducted a randomized controlled experiment (N = 40) to evaluate the role of sex as a potential moderator [42]. Half of the sample were males and half were females, all of which completed two counterbalanced visits (exercise and no exercise). The exercise visit included a 15-minute bout of moderate intensity treadmill exercise, while the control visit included a 15-minute seated task. Participants completed the RAVLT as the memory assessment after the bout of exercise. A repeated measures ANOVA indicated that, when examining List B outcomes, there was a main effect for condition (*p =* 0.02), but there was no sex by condition interaction (*p =* 0.23). When examining Trial 1—List B, there was, again, no sex by condition interaction (*p =* 0.14), but there was a main effect for condition (*p =* 0.02). These findings suggest that acute moderate intensity exercise was beneficial for reducing memory interference, but the effect was not influenced by biological sex. 

To summarize the results of our previous studies, exercising prior to a memory interference task tends to improve memory outcomes by lessening the memory interference effect. There is some evidence that exercise may attenuate both a proactive and retroactive interference effect, but further research is still needed, especially surrounding potential moderators of the relationship, as there are individual differences that may impact the relationship between exercise and memory interference, such as age or depression symptomology [43]. Further work should also consider whether the memory interference protocol (e.g., RAVLT, AB/AC) influences the effects of exercise on attenuating memory interference, as they differ in their effectiveness, structure, and ability to measure PI and/or RI. 

Animal studies demonstrate some evidence of exercise improving memory function and attenuating memory interference. In a study by Bolz (2015), mice were divided into the following groups for several weeks: sedentary, voluntary running, and voluntary running within an enriched environment. The mice were subsequently exposed to a Novel Object Recognition (NOR) task to examine if there was a measurable difference in pattern separation (the importance of which will be addressed later) [44]. Mice were exposed to two identical objects (cones or pyramids) within their arena for a six-minute sample phase. Subsequently, one of the objects was replaced with a new, different object and memory was assessed by comparing time spent exploring the novel object with time spent examining the familiar object. This testing phase lasted five minutes, with delayed testing phases occurring after 1.5 hours and 24 hours. The results from this study demonstrated three main findings. First, voluntary running increased the number of, and dendritic length of, newly generated young neurons, with running mice demonstrating a four-fold increase in the number of young granule cell dendrites [44]. Second, running improved pattern separation during novel object recognition, as observed by time spent exploring novel versus familiar objects. The final result from the study demonstrated that exercise inhibited temporal decay of pattern separation after learning [44]. The object recognition memory for mice in the control condition decayed within 24 hours, whereas the voluntary running mice, in both conditions, maintained their memory after a 24-hour delay period [44]. A potential mechanism of this significant difference is the exercise-induced increase in new synapses and dendrites [44]. Overall, running demonstrated a meaningful improvement of hippocampal (functional connectivity) pattern separation in regard to object recognition, and thus, attenuated a memory interference effect [45]. Notably, however, these findings may need to be interpreted cautiously as it was unclear how distinct the new and old objects were. The perirhinal cortex also plays a significant role in object recognition tasks, as it assists with recognition memory [46,47]. Exercise-induced changes in BDNF levels within the perirhinal cortex have also been demonstrated, along with increases in functional connectivity, afferent input to the hippocampus, and short-term synaptic plasticity [48,49,50,51]. Therefore, both the perirhinal cortex and the hippocampus may play a role in attenuating a memory interference effect.

## 10. Mechanisms of Attenuating Memory Interference

**Figure 4:**
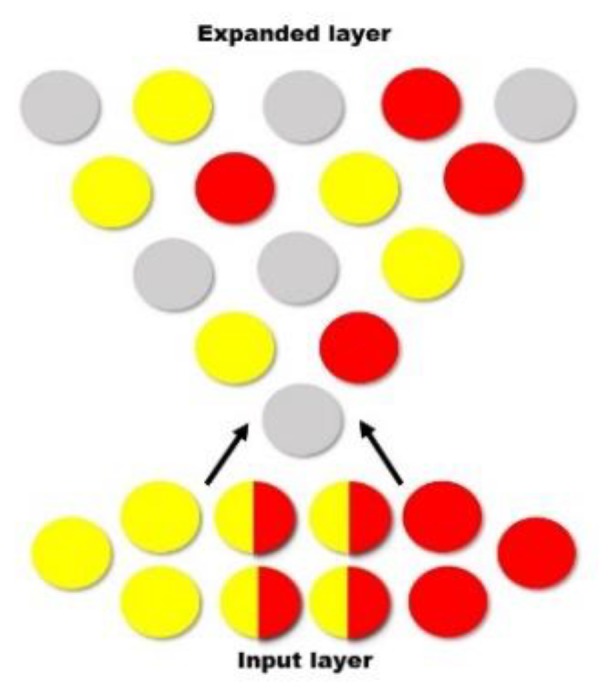
When List 1 (yellow) and List 2 (red) are originally encoded, there is an initial overlap. When the engram is integrated into a larger population of engrams, the original engrams are distributed (among already established memories—grey) to minimize overlap.

**Figure 5:**
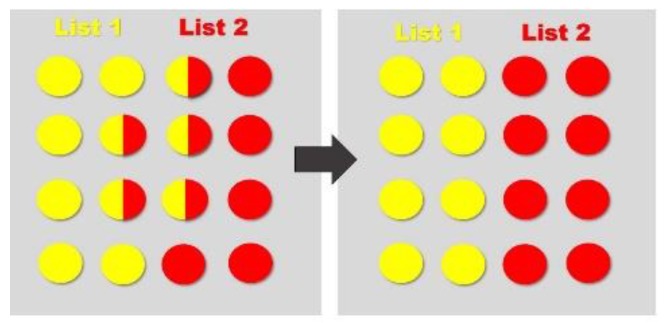
Learning of List 1 (yellow) and List 2 (red) creates an overlapping engram due to similarities in context. Pattern separation occurs, distinguishing List 1 from List 2.

**Figure 6:**
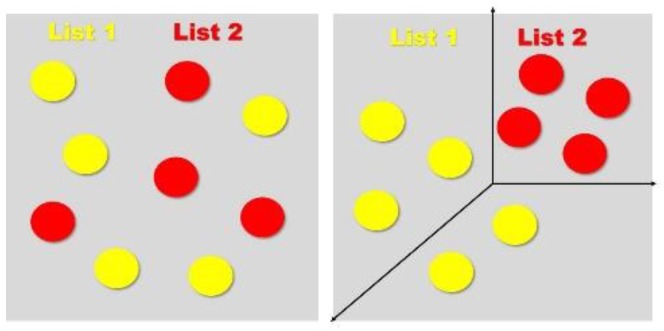
List 1 (yellow) and List 2 (red) engrams seem to overlap from one angle, but a third neural circuit facilitates pattern separation by expanding the dimensionality.

When competing stimuli are encoded, for example by completing an AB/AC paired-associative learning task and learning two lists of similar word pairs (e.g., house—table, house—sugar) [14], there is overlap within the memory trace—see Figure 4. When the engram is eventually integrated into a larger population of engrams, during consolidation, the original engrams are separated, minimizing overlap, and in turn, create two distinct engrams [35]. When memories are encoded within the hippocampus, the medial prefrontal cortex (mPFC) reduces memory interference by distinguishing patterns and separating them accordingly—see Figure 5 [35]. Within an engram, independent neural circuits may also facilitate pattern separation from a three-dimensional point of view—see Figure 6. That is, and as indicated elsewhere [35], certain brain structures, such as the cerebellar cortex, induce pattern separation by expanding the dimensionality, enabling a downstream decoder neuron to linearly classify them. This pattern separation is thought to facilitate associative learning by making the neural representations more distinct, ultimately making the unconditioned stimulus less likely to be activated mistakenly [52]. Figure 4, Figure 5 and Figure 6 have been adapted from Cayco-Gajic and Silver [52].

Pattern separation is vital to reducing memory interference. Mechanistically, there is evidence of communication between the amygdala and the hippocampus in assisting with pattern separation of emotional memories [53]. Further, in addition to the amygdala and hippocampus working in concert to facilitate pattern separation, and in addition to the medial prefrontal cortex, recent work demonstrates that the lateral prefrontal cortex and hippocampus also work together to assist in pattern separation [54]. The hippocampus may be a facilitator of pattern separation, particularly temporal pattern separation (i.e., elapsed period of time), while the amygdala moderates the strength of the specific memory [53,55]. Using intracranial recordings, brain waves, measured utilizing specific oscillatory modes of frequency, directionality, and phase information, may facilitate pattern separation specifically through theta wave oscillations [53]. That is, coordinated theta wave oscillations between the amygdala and hippocampus may help facilitate pattern separation of emotional information. These findings support a bidirectional relationship between the amygdala and hippocampus in supporting pattern separation [53]. Exercise has been demonstrated to not only increase theta wave activity [56], but also increase neural activity within the amygdala and hippocampus [57,58,59]. 

Other research has also demonstrated that the dentate gyrus, a region within the hippocampus, uses pattern separation to process spatial and episodic memories, as animals with a damaged dentate gyrus have a diminished ability to distinguish between two similar objects [60,61]. The dentate gyrus contains over four times more neurons than upstream (entorhinal cortex) or downstream (CA3) pathways [62]. Input from relatively fewer cells is processed by a much larger neural network in the dentate gyrus before a condensed output is generated. As such, the dentate gyrus functions as a pattern separator by partially de-correlating inputs [63,64]. Although most of this research has been conducted on animals, there is also evidence of increased dentate gyrus activity during pattern separation activities in humans [60]. Exercise may also help facilitate pattern separation through increased neural activity in the dentate gyrus [45]. Adult neurogenesis, which occurs in this region of interest, is associated with an increased ability to distinguish patterns, as when neurogenesis is ablated in mice, performance severely decreases in pattern separation tasks [60]. Adult neurogenesis is also increased by exercise, providing another means through which exercise may attenuate a cognitive memory interference effect [60,65]. In addition to exercise-induced neural changes in the dentate gyrus, and exercise-induced neurogenesis in the hippocampus, exercise may also facilitate pattern separation by altering limbic tract integrity [66,67]. That is, exercise may facilitate white matter integrity, which has been shown to play a critical role in pattern separation, supporting the notion that pattern separation relies on broad neural networks that connect to the hippocampus [68]. One such network involves the mPFC. Other work, such as that by Frankland, Kohler, and Josselyn (2013), examined a different perspective of how neurogenesis may affect memory interference. This case suggests that ongoing neurogenesis produces retroactive interference regardless of content [69]. These authors predict that, in animal studies, increasing hippocampal neurogenesis should weaken existing hippocampal memories, whereas decreasing hippocampal neurogenesis should have the opposite effect and protect existing memories within the hippocampus [69]. 

The mPFC also influences memory interference [31,35]. Patients with prefrontal lobe damage perform as well as their healthy counterparts on basic memory tasks, yet exhibit poor performance on paired associate learning tasks and other memory interference tasks (e.g., RAVLT), suggesting that the mPFC is necessary for memory interference [31,35]. In a rat model, Guise and Shapiro (2017) demonstrated that the completion of a plus maze task requires both the hippocampus and mPFC [35]. When the mPFC was inactivated, rats were still able to learn spatial cues and retrieve memories, but they were incapable of task switching—learning new responses to previously associated and learned cues [35]. In other words, an inactive mPFC impaired the rats’ ability to change spatial rules (e.g., turn left at the fork instead of right), suggesting that the mPFC sends task-specific rules to the hippocampus—distinguishing between competing memories and retrieving the relevant one [35]. 

**Figure 7:**
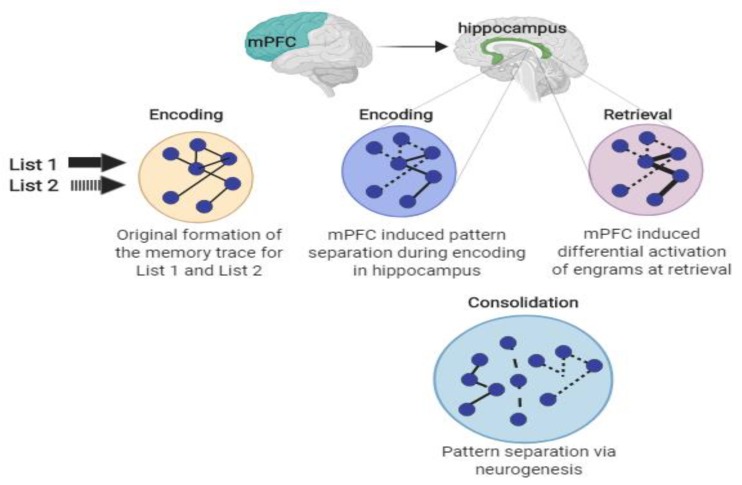
Memory interference attenuation. Memory interference attenuation via mechanisms related to pattern separation, differential activation, and neurogenesis. The learning of List 1 and List 2 creates a shared memory trace (engram). The mPFC induces pattern separation during encoding within the hippocampus, creating distinct patterns for each list (dotted versus solid line). During retrieval of List 1, the mPFC differentially activates the corresponding memory trace (represented by the bold line). Lastly, exercise-induced neurogenesis may also induce pattern separation by the creation of new neurons between the existing traces (represented by the new dashed lines).

Figure 7 schematically illustrates the integral role of the mPFC in attenuating memory interference. Upon encoding of similar material (e.g., AB/AC paired associate learning task), the original engram has initial overlap. The mPFC induces pattern separation within the hippocampus, creating a distinguishing pattern between List 1 and List 2, similar to Figure 4; Figure 5. During retrieval of a target list, the mPFC also differentially activates the relevant list, shown in the figure by a bolded line versus a dotted line. The final hypothesized mechanism is through neurogenesis, which we discussed previously, where the creation of new neurons may separate established engrams, removing the initial overlap. 

Additionally, the ventral lateral prefrontal cortex (VLPFC) and dorsal lateral prefrontal cortex (DLPFC) are also found to be involved in memory interference, especially proactive interference in working memory [70]. Dulas et al. [70] investigated age-related changes in overcoming proactive interference in associative memory, which found that under conditions of high interference, older adults showed reduced associative memory accuracy effects in the DLPFC and anterior PFC. Both lesion [71] and transcranial magnetic stimulation studies (TMS) [72] observed that disrupting the left VLPFC results in both increased errors and increased response time in proactive interference tasks. Some fMRI studies suggest that older adults show reduced sensitivity to proactive interference in working memory [73]. Therefore, one study examined older adults’ proactive interference, which showed that the activity of left mid-VLPFC increased with increasing interference level in both correct associative memory responses and incorrect associative memory responses during proactive interference [70]. Taken together, although it remains largely unknown, mPFC, VLPFC and DLPFC may play different roles in memory interference. 

## 11. Hypothesized Mechanisms through Which Aerobic Exercise May Attenuate Memory Interference

**Figure 8:**
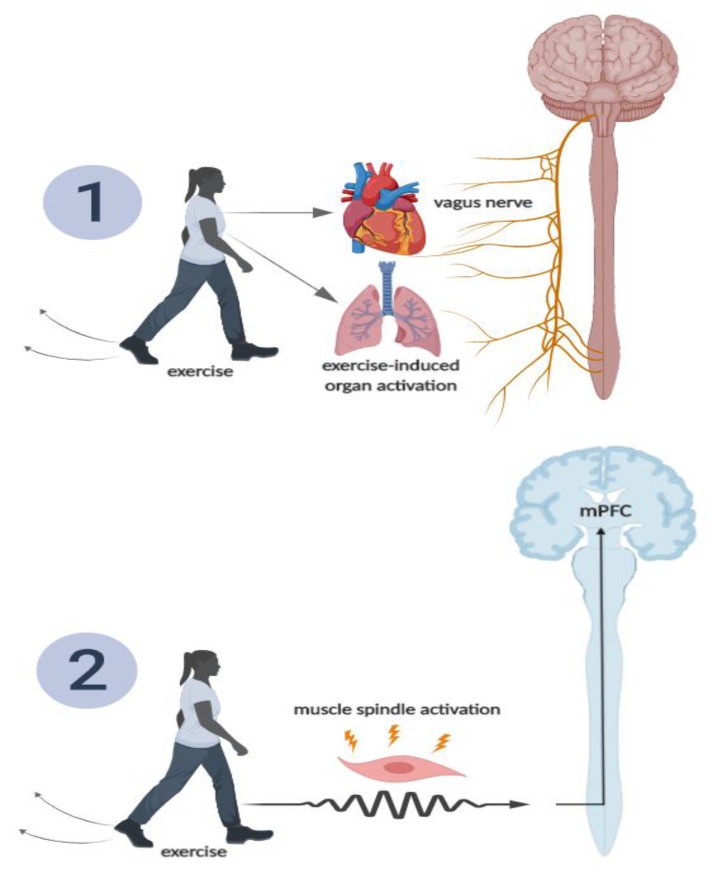
Hypothesized schematic of two routes through which acute exercise may activate the medial prefrontal cortex (mPFC), and in turn, attenuate memory interference. Pathway 1 includes exercise-induced skeletal muscle spindle activation, which activates peripheral nerves, sending action potentials to the brainstem, ultimately activating the mPFC. Pathway 2 includes exercise-induced activation of the mPFC via vagus nerve stimulation (e.g., afferent vagus nerve stimulation from lung expansion and increased myocardial contractility, as well as increased catecholamine production) [14].

There are multiple hypothesized pathways through which acute (single bout) exercise may activate these key brain regions involved in attenuating memory interference, two of which we detail in the following section. The first pathway involves the activation of the vagus nerve (see Figure 8, pathway 1). The vagus nerve (afferent fibers) may become stimulated by activation of various tissues during exercise (e.g., heart, lungs) or exercise-induced increases in catecholamines [29]. Afferent sensory fibers of the vagus nerve send information from the peripheral tissues to the nucleus of the tractus solitarius (NTS), which again projects directly to the hippocampus and PFC [29]. Through the muscle spindle pathway [1,2,3] (see Figure 8, pathway 2), skeletal muscles are activated during exercise, causing muscle spindles to become activated, which, in turn, generate action potentials [29]. These action potentials are transmitted by afferent peripheral nerves and are sent to the spinal cord and brain stem [29]. Within the brain stem, the NTS has a direct projection to the prefrontal cortex and locus coeruleus (LC) [12,29]. The PFC, again, plays a major role in pattern separation [29], while the LC has direct projections to hippocampal structures, which have also been established to influence pattern separation [53]. These described routes detail the anatomical pathway through which exercise may, although they need to be more thoroughly investigated, increase brain activity in key memory-related brain structures (e.g., hippocampus and PFC), thereby potentially facilitating the attenuation of memory interference and improving episodic memory function.

In summary, memory is imperative for daily functioning, and methods to reduce memory impairment should be continuously pursued. Memory interference is a candidate mechanism as to why information is forgotten. Several brain regions (e.g., hippocampus, mPFC and amygdala) are involved in pattern separation, which are vital for reducing memory interference. Exercise may help to facilitate memory function and attenuate memory interference via several potential mechanisms, including, for example, increased neural activity in key brain regions involved in pattern separation (e.g., hippocampus, mPFC), increased neurogenesis in these brain regions, and increased functional connectivity across these brain regions by enhancing white matter integrity in the tracts that connect these structures. 

## 12. Future Directions 

Due to its scarcity, there is a broad range of future directions for memory interference research to explore. First and foremost, future work should confirm the potential hypothesized mechanisms and pathways that are mentioned in this review. More human based, in addition to rodent, experiments need to be conducted. Investigating the hypothesized pathways of exercise-induced activation of muscle spindles and the vagus nerve would allow for further expansion on the mechanisms behind exercise’s ability to improve memory function (other feedback pathways, such as those coming from skin and joint receptors, should also be considered). As stated previously, Guise and Shapiro detail the relationship between the hippocampus and mPFC and the importance of mPFC activation for a task-switching task. Due to its strong implications in memory interference, more work is also required to examine this relationship of distinguishing between competing information and identifying the relevant information in human studies. Moreover, identifying respective functions among DLPFC, VLPFC as well as mPFC could deepen our understanding of the neural correlates underlying memory interference. Within the current body of human research evaluating the effects of acute exercise on memory interference, most studies have focused on short-term memory. Thus, future research should evaluate this topic using long-term memory interference protocols. Additional work should evaluate whether acute exercise can attenuate memory interference when the acute bout of exercise occurs during the memory consolidation phase. Further, most of the human studies on this topic have focused on an acute exercise paradigm, and as such, additional work should evaluate the effects of chronic exercise on memory interference. Some emerging work suggests that habitual exercise engagement is associated with better pattern separation on a visual-based mnemonic similarity task [43]. Lastly, given that there are notable individual differences in memory function, future work should evaluate whether individual differences in memory moderates the effects of exercise on memory interference. These directions, although few, will strengthen the field and expound our knowledge of the relationship between exercise and memory interference. 

## Abbreviations

BDNF	brain derived neurotrophic factor
DLPFC	dorsolateral prefrontal cortex
IGF-1	insulin-like growth factor 1
LC	locus coerulous
mPFC	medial prefrontal cortex
MRI	magnetic reasoning imaging
NTS	nucleus of the tractus solitarius
PI	proactive interference
RAVLT	Rey auditory verbal test
RI	retroactive interference
VEGF	vascular endothelial growth factor
VLPFC	ventrolateral prefrontal cortex
VO_2_ max	maximal oxygen uptake
